# Identification of Key Pathways and Genes of Acute Respiratory Distress Syndrome Specific Neutrophil Phenotype

**DOI:** 10.1155/2019/9528584

**Published:** 2019-08-19

**Authors:** Dong Wang, Yajuan Li, Changping Gu, Mengjie Liu, Yuelan Wang

**Affiliations:** ^1^Department of Anesthesiology, Shandong Provincial Qianfoshan Hospital, the First Hospital Affiliated with Shandong First Medical University, Jinan 250014, China; ^2^Department of Anesthesiology, Taian City Central Hospital, Taian 271000, China

## Abstract

Despite over 50 years of clinical and basic studies, acute respiratory distress syndrome (ARDS) is still a critical challenge with high mortality worldwide. The severity of neutrophil activation was associated with disease severity. However, the detailed pathophysiology of the circulating polymorphonuclear neutrophil activation in ARDS remains unclear. To identify key pathways and genes in the ARDS-specific neutrophil phenotype distinct from sepsis, the datasets of blood polymorphonuclear neutrophils (PMNs) from patients with ARDS (GSE76293) and from sepsis patients (GSE49757) were chosen from the Gene Expression Omnibus (GEO) and analyzed using bioinformatics methods. A total of 220 differential expressed genes (DEGs) were overlapped between GSE49757 and GSE76293 in a Venn diagram. Pathway enrichment analysis results showed that DEGs in GSE76293 were mainly enriched in the MAPK signaling pathway, FoxO signaling pathway, and AMPK signaling pathway relative to GSE49757. We identified 30 hub genes in the protein-protein interaction network. By comparing with GSE49757, we speculated that GAPDH, MAPK8, PIK3CB, and MMP9 may play important roles in the progression of ARDS-specific circulating neutrophil activation. The findings may provide novel insights into the development of promising targets for the diagnosis and treatment of ARDS in the future.

## 1. Introduction

Acute respiratory distress syndrome (ARDS) is characterized by diffuse damage of the alveolar-capillary barrier, immune cell infiltration, protein-rich edema fluid in the alveoli, and severe gas-exchange abnormalities. Despite over 50 years of clinical and basic studies, ARDS is still a critical challenge with high mortality worldwide. ARDS increases healthcare costs and impairs quality of life seriously [1]. Therefore, getting a better understanding of the pathogenesis of ARDS is urgent and highly demanded.

Polymorphonuclear neutrophils (PMNs) are crucial for controlling infections as innate immune system cells [2]. Circulating PMNs become activated and penetrate the alveolar-capillary barrier into the airspaces in the progression of ARDS. PMNs in the alveoli inflammatory microenvironment become further activated to play an important role in phagocytosing pathogens, releasing reactive oxygen species and inducing neutrophil extracellular traps [3–6]. Subsequently, activated neutrophils lead to alveolar damage and further loss of lung function. However, the mechanisms of blood PMNs activation and infiltration in the development of ARDS remain poorly understood.

High-throughput gene profiling has been a powerful tool in revealing key pathways and genes of lung disease, such as ARDS and asthma [7, 8]. The transcriptomics mining of key pathways and genes offers a potential direction for future mechanism research. One of the major issues in previous high-throughput studies of ARDS was that samples were whole blood or total leukocytes rather than purified neutrophils. In the present study, we identified differential expressed genes (DEGs) in blood PMNs from patients with ARDS (GSE76293) and from sepsis patients (GSE49757) and used integrated bioinformatics methods to identify key pathways and genes in the ARDS-specific neutrophil phenotype distinct from sepsis. Our findings may provide novel potential targets for the diagnosis and treatment of ARDS.

## 2. Materials and Methods

### 2.1. Microarray Data

To investigate the ARDS-specific neutrophil phenotype distinct from sepsis, we searched expression profiles of ARDS blood PMNs and chose the datasets GSE76293 [9] and GSE49757 [10] from the Gene Expression Omnibus (GEO) [11]. In the current study, 12 ARDS blood PMNs samples and 12 HVT blood PMNs samples were used for analysis in the dataset GSE76293; 20 PMNs samples stimulated with severe sepsis plasma and 19 PMNs samples stimulated with HVT plasma were selected to verify the ARDS-specific neutrophil phenotype in the dataset GSE49757.

### 2.2. Identification of DEGs

DEGs were identified using GEO2R (http://www.ncbi.nlm.nih.gov/geo/geo2r/), which is an online tool based on the GEOquery and Limma R packages [12]. The genes that met the cut-off criteria of an adjusted* p* value < 0.01 and a |log2 fold change| > 0.585 were considered DEGs. To indicate the intersection among DEGs between GSE49757 and GSE76293, a Venn diagram was produced by a Venn webtool (http://bioinformatics.psb.ugent.be/webtools/Venn/).

### 2.3. Gene Ontology (GO) and Pathway Enrichment Analyses

The Database for Annotation Visualization and Integrated Discovery (DAVID; http://david.ncifcrf.gov, version 6.7) [13, 14] was used to analyze the gene ontology and pathway of DEGs as previously described [15, 16].* p* value < 0.05 was used as a threshold to define significantly enriched terms.

### 2.4. Protein-Protein Interactions (PPI) Network and Module Analysis

The Search Tool for the Retrieval of Interacting Genes (STRING; http://string-db.org, version 10.5) online database was used to predict interactions of DEGs [17]. A PPI network was drawn by Cytoscape (version 3.7.1). Furthermore, the plugins CytoNCA [18] and MCODE [19] of Cytoscape were used to identify the hub genes and modules in the PPI network.

### 2.5. Transcription Factor (TF) Regulatory Network Analysis

The iRegulon plugin in Cytoscape was used to predict TFs of the selected hub DEGs [20]. A normalized enrichment score (NES) >4 was considered the threshold value.

### 2.6. Relative mRNA Expression Level of Hub Genes

To verify the differences of relative mRNA expression level of hub genes between GSE49757 and GSE76293, we downloaded the matrix data of the two datasets from the GEO and analyzed the log2 normalized signal intensity of selected hub genes using GraphPad Prism 7.04.

### 2.7. Statistical Analysis

All statistical analyses in this study were performed using GraphPad Prism 7.04 (GraphPad Software, San Diego, CA, USA), and* p* < 0.05 was considered to be significant. Data are presented as mean ± SEM. Student's* t*‐test was used to compare difference.

## 3. Results

### 3.1. Identification of DEGs

**Figure 1:**
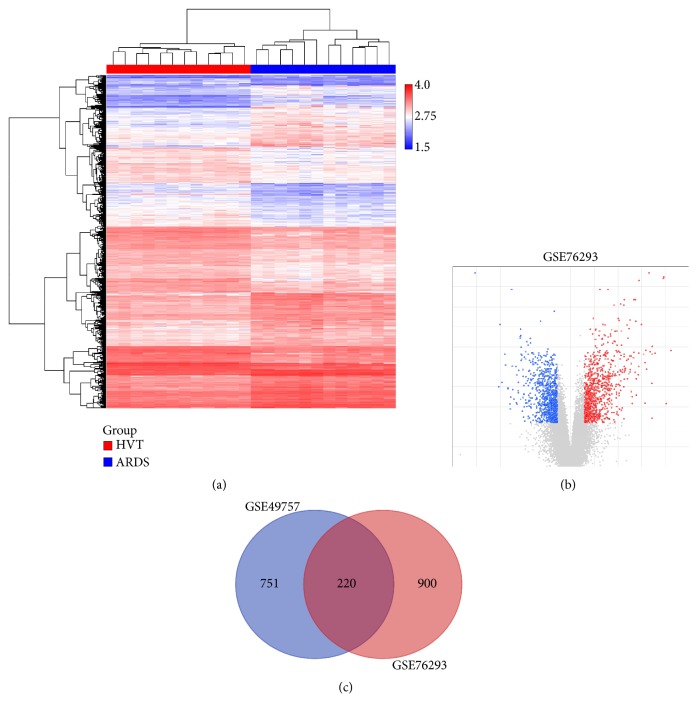
Hierarchical clustering, volcano plot, and Venn diagram of differentially expressed genes in GSE49757 and GSE76293. Hierarchical clustering indicates the gene expression profile of GSE76293 (a): red to blue colors refer to be high to low relative expression levels. Volcano plot of differentially expressed genes in GSE76293 (b): gray dots indicate no change; blue and red dots indicate downregulated and upregulated genes, respectively. Venn diagram indicates the intersection among DEGs between GSE49757 and GSE76293 (c).

In total, 1120 mRNAs were significantly differentially expressed in ARDS blood PMNs compared with HVT blood PMNs, including 486 upregulated genes and 634 downregulated genes (Figures 1(a) and 1(b)). There were 971 DEGs in PMNs exposed to severe septic plasma compared with unstimulated controls, including 455 upregulated genes and 516 downregulated genes. A total of 220 genes were overlapped between GSE49757 and GSE76293 in the Venn diagram (Figure 1(c)).

### 3.2. GO and Pathway Enrichment Analyses

**Figure 2:**
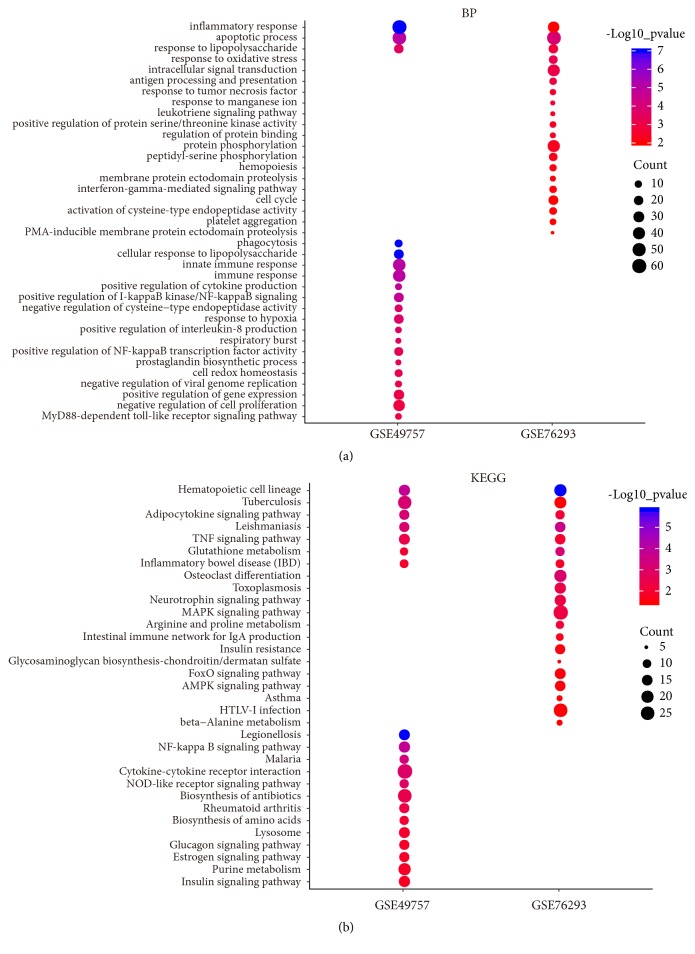
Biological process (BP) (a) and pathway enrichment (b) of differentially expressed genes in GSE49757 and GSE76293 (top 20,* p* < 0.05). Red to blue colors indicate low to high -log10 (*p* value) levels. Point size indicates the number of differentially expressed genes in the corresponding items.

DEGs in GSE76293 were mainly associated with the following biological processes: apoptotic process, response to oxidative stress, response to lipopolysaccharide, response to tumor necrosis factor, and leukotriene signaling pathway (Figure 2(a)). The results also indicated that DEGs in GSE76293 were mainly enriched in the following pathways: MAPK signaling pathway, FoxO signaling pathway, AMPK signaling pathway, and TNF signaling pathway (Figure 2(b)).

DEGs in GSE49757 were mainly associated with the following biological processes: inflammatory response, response to lipopolysaccharide, apoptotic process, immune response, positive regulation of cytokine production, and positive regulation of NF-kappaB signaling (Figure 2(a)). The results also indicated that DEGs in GSE49757 were mainly enriched in the following pathways: NF-kappa B signaling pathway, cytokine-cytokine receptor interaction, NOD-like receptor signaling pathway, and TNF signaling pathway (Figure 2(b)).

### 3.3. PPI Network Analysis

**Figure 3:**
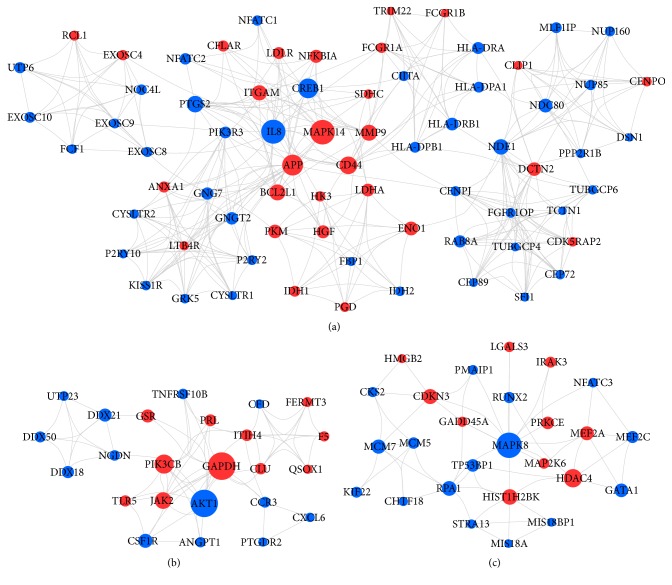
The three most significant modules of the PPI network in GSE76293. Red and blue circles indicate upregulated and downregulated differentially expressed genes, respectively. Circle size indicates the node degree.

The PPI network of 899 nodes with 3757 protein interaction pairs was constructed to identify hub genes in ARDS circulating PMN activation in GSE76293. The top 30 hub genes with the higher degree were listed in Table 1. The three most significant modules of the PPI network were shown in Figure 3, in which GAPDH, AKT1, MAPK14, MAPK8, IL8, PIK3CB, and MMP9 were the top hub genes.

### 3.4. TF Regulatory Network Analysis

**Figure 4:**
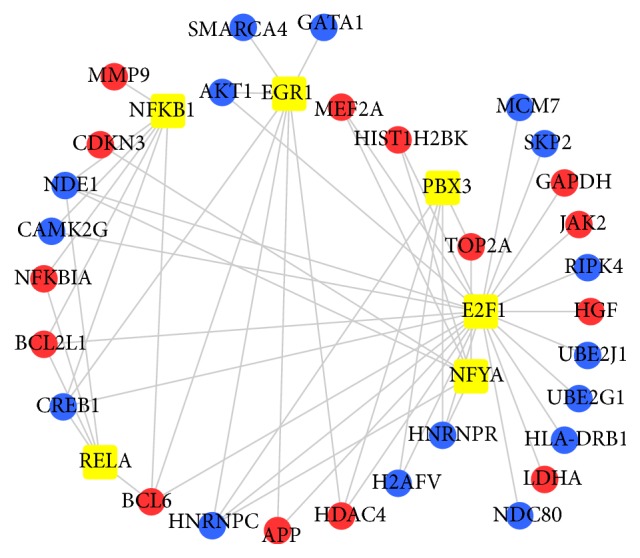
TF regulatory network of the top 50 hub genes in the PPI network in GSE76293. Yellow squares indicate TFs. Red and blue circles indicate upregulated and downregulated differentially expressed genes, respectively.

The TFs which regulated the top 50 hub genes in the PPI network were predicted. With a threshold of an NES > 4, a total of six TFs (E2F1, NFKB1, NFYA, PBX3, EGR1, and RELA) were revealed to be associated with 30 target hub genes in Figure 4.

### 3.5. Relative mRNA Expression Level of Hub Genes

**Figure 5:**
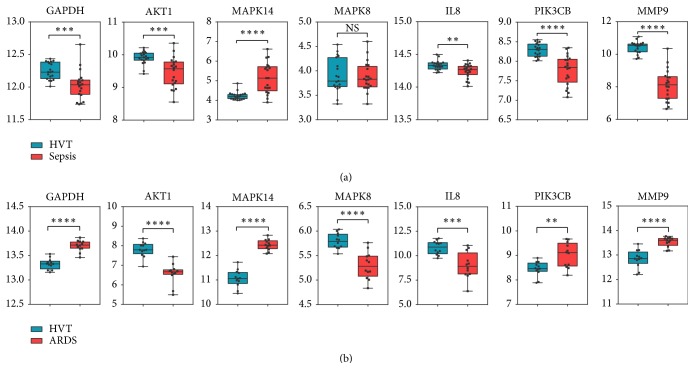
The relative mRNA expression level of the seven hub genes in GSE49757 (a) and GSE76293 (b). All data are means ± SEM, Student's* t*-test. ^*∗∗*^*p* < 0.01; ^*∗∗∗*^*p* < 0.001; ^*∗∗∗∗*^*p* < 0.0001; NS, not significant.

To identify the hub genes of ARDS-specific neutrophil phenotype distinct from sepsis, we compared the relative mRNA expression levels of selected hub genes in both GSE49757 and GSE76293. We found that AKT1 and IL8 were downregulated, while MAPK14 was upregulated in both GSE49757 and GSE76293 (Figure 5). At the same time, the relative expression trends of GAPDH, MAPK8, PIK3CB, and MMP9 were different between GSE49757 and GSE76293 (Figure 5).

## 4. Discussion

Acute respiratory distress syndrome (ARDS) is characterized by diffuse damage of the alveolar-capillary barrier, immune cell infiltration, protein-rich edema fluid in the alveoli, and severe gas-exchange abnormalities. The severity of neutrophil activation and infiltration was associated with disease severity [21, 22]. However, the detailed pathophysiology of the ARDS-specific neutrophil activation remains unclear, especially the complicated molecular mechanisms.

The rapid development of high-throughput detection technology in recent decades has provided technical support for genome-wide analyses of changes in gene expression related to ARDS. However, one of the issues in previous high-throughput studies of ARDS was that samples were whole blood or total leukocytes rather than purified neutrophils. In the present study, we chose the dataset GSE76293 including 12 ARDS blood PMNs samples and 12 HVT blood PMNs samples. There were 1120 DEGs in ARDS blood PMNs compared with HVT controls, including 486 upregulated mRNAs and 634 downregulated mRNAs.

Because not all patients with severe sepsis develop ARDS, a comparison of circulating neutrophils from patients with ARDS with neutrophils from severe septic patients may allow us to find the ARDS-specific neutrophil phenotype distinct from sepsis. Therefore, we searched sepsis blood neutrophils expression profile in the GEO and chose the dataset GSE49757, in which 20 PMNs samples stimulated with severe septic plasma and 19 PMNs samples stimulated with HVT plasma samples were used for analysis. There were 971 DEGs between severe septic plasma samples and controls, including 455 upregulated genes and 516 downregulated genes. The overlap between GSE49757 and GSE76293 contained 220 genes in the Venn diagram.

DEGs in GSE76293 were mainly enriched in the following biological processes: response to oxidative stress, leukotriene signaling pathway, and response to tumor necrosis factor compared with GSE49757. KEGG enrichment analysis results showed that DEGs in GSE76293 were mainly involved in the MAPK signaling pathway, FoxO signaling pathway, and AMPK signaling pathway relative to GSE49757. These findings, while preliminary, suggested that these biological processes and pathways played important roles in the ARDS-specific neutrophil phenotype distinct from sepsis.

To identify hub genes involved in the activation of circulating PMNs in ARDS, we constructed the PPI network and predicted its key modules. We found that GAPDH, AKT1, MAPK14, MAPK8, IL8, PIK3CB, and MMP9 were the top hub genes in the three most significant modules. We speculated that the seven hub genes may affect the circulating PMN activation in ARDS. To further identify whether the seven hub genes were involved in the ARDS-specific neutrophil phenotype distinct from sepsis, we verified the relative mRNA expression of the seven genes in GSE49757. We found that AKT1 and IL8 were downregulated; MAPK14 was upregulated in both GSE49757 and GSE76293. However, the relative expression trends of GAPDH, MAPK8, PIK3CB, and MMP9 were different between GSE49757 and GSE76293. We speculated that GAPDH, MAPK8, PIK3CB, and MMP9 may play important roles in the ARDS-specific neutrophil phenotype distinct from sepsis.

Phosphoinositide 3-kinases (PI3Ks) participate in most pathophysiology processes in almost all human tissues. PIK3CB played an important role in neutrophil survival, priming, activation, and ROS production [23–26]. Previous reports have shown that AKT was one of the important effectors of PI3K signaling [27]. PIK3CB resulted in the phosphorylation of AKT; in turn, AKT activation participated in the activation of downstream effectors of the PI3K pathway. Our results showed that PIK3CB was upregulated, whereas AKT1 was significantly downregulated in ARDS blood PMNs compared with HVT blood PMNs. We speculated that one possible explanation for these results may be that the PI3K pathway interacts with other signaling pathways and forms complex interaction networks, which cause the specific cellular response.

Mitogen‐activated protein kinases (MAPKs) are involved in most of the cellular responses to harmful stimuli (like infection, oxidative stress, etc.) [28–31]. One interesting finding was that MAPK14 was significantly upregulated while MAPK8 was downregulated in ARDS blood PMNs compared with HVT blood PMNs. It is difficult to explain this result, but it might be related to the functional differences in apoptosis between MAPK14 and MAPK8. Prior studies have noted that circulating neutrophils from patients with ARDS exhibited delayed apoptosis. Phosphorylation of MAPK14 led to the inhibition of neutrophil apoptosis [32], while MAPK8 induced apoptosis or growth inhibition [33, 34]. We speculated that the differential expression of MAPK14 and MAPK8 may contribute to the delayed apoptosis of circulating neutrophils.

In patients with ARDS, immunocytes such as macrophages in the regions of pulmonary injury secrete chemokines, of which IL-8 is the typical neutrophil chemokine [5, 35]. Previous studies have shown that IL-8 was elevated in bronchoalveolar lavage fluid PMNs from patients with ARDS. Here, we noticed that IL-8 was downregulated in ARDS blood PMNs compared with HVT controls in GSE76293. In addition, IL-8 expression was also downregulated in PMNs exposed to severe septic plasma compared to unstimulated controls in GSE49757. The reason for this is not clear, but one possible explanation for these results may be that the downregulation of IL-8 contributes to circulating neutrophil recruitment into the regions rich in IL-8.

Glyceraldehyde-3-phosphate dehydrogenase (GAPDH) is a key enzyme in the process of glycolysis. GAPDH is often considered a housekeeping gene and a control for western bolt and qPCR because of the stable and high expression in most cells and tissues. However, increasing studies have reported that GAPDH was associated with many physiological functions, such as inflammatory and immune responses [36–38]. Piszczatowski RT et al. found that myeloid zinc finger-1 (MZF-1) regulated the translation of GAPDH [39]. Here, we found that GAPDH was upregulated in ARDS blood PMNs compared with HVT blood PMNs in GSE76293 and might be regulated by the E2F1 transcription factor [40]. In addition, the present study showed that GAPDH was downregulated in PMNs exposed to severe septic plasma relative to unstimulated controls in GSE49757. Therefore, it needs to be considered with caution when GAPDH is used as a control for qPCR in future studies.

Despite the results obtained above, there were some limitations in this study. Because of the relatively small sample size and the heterogeneity of ARDS, the findings need to be interpreted with caution. The results need to be further validated in a large number of samples, and the mechanisms need to be investigated both in vitro and vivo in the future.

## 5. Conclusions

In conclusion, we identified key pathways and genes involved in the ARDS-specific neutrophil phenotype distinct from sepsis. We speculated that GAPDH, MAPK8, PIK3CB, and MMP9 may play important roles in the progression of ARDS-specific circulating neutrophil activation. The findings may provide novel insights into the development of promising targets for the diagnosis and treatment of ARDS in the future.

## Figures and Tables

**Table 1:** The top 30 hub genes of the PPI network in GSE76293. logFC: log2 fold change between two experimental conditions. adj.P.Val: *p* value after adjustment for multiple testing.

Gene symbol	Degree	logFC	adj.P.Val
GAPDH	106	1.28	2.69E-06
AKT1	105	-1.23	6.93E-05
RIPK4	102	-1.62	3.42E-04
TOP2A	87	0.97	3.44E-03
MAPK14	69	1.50	1.74E-07
MAPK8	68	-0.97	2.15E-03
CXCL8	67	-2.12	1.97E-03
SMARCA4	66	-0.62	7.63E-03
PIK3CB	60	0.65	3.56E-03
APP	55	1.37	4.14E-03
CREB1	51	-0.68	6.18E-03
JAK2	47	0.95	5.29E-05
CD44	43	1.82	2.85E-06
HDAC4	42	0.97	1.66E-05
PTGS2	39	-1.47	5.15E-03
H2AFV	39	-0.68	2.36E-03
MMP9	37	0.69	2.75E-04
BCL2L1	37	0.84	3.26E-03
SKP2	36	-0.69	5.53E-03
POLR2I	35	-0.69	2.77E-03
NDE1	35	-0.72	8.75E-04
ITGAM	34	0.79	5.92E-05
SNU13	34	-0.78	3.66E-03
CSF1R	33	-2.10	1.08E-05
HIST1H2BK	32	0.60	1.05E-04
MIB2	31	-0.73	5.11E-04
CDKN3	30	0.89	6.48E-04
UBE2D4	29	-0.59	1.51E-03
NDC80	29	-1.13	6.52E-04

## Data Availability

The data used to support the findings of this study are available from the corresponding author upon request.
